# Metabolomics applied to maternal and perinatal health: a review of new frontiers with a translation potential

**DOI:** 10.6061/clinics/2019/e894

**Published:** 2019-03-14

**Authors:** Renato Teixeira Souza, Jussara Mayrink, Débora Farias Leite, Maria Laura Costa, Iracema Mattos Calderon, Edilberto Alves Rocha, Janete Vettorazzi, Francisco Edson Feitosa, José Guilherme Cecatti, Preterm SAMBA Study Group

**Affiliations:** I Departamento de Ginecologia e Obstetricia, Faculdade de Ciencias Medicas, Universidade Estadual de Campinas, Campinas, SP, BR.; II Departamento Materno Infantil, Faculdade de Medicina, Universidade Federal de Pernambuco, Pernambuco, PE, BR.; III Departamento de Ginecologia e Obstetricia, Faculdade de Medicina de Botucatu, Universidade Estadual de Sao Paulo (UNESP), Botucatu, SP, BR.; IV Departamento de Ginecologia e Obstetricia, Faculdade de Medicina, Universidade Federal do Rio Grande do Sul, Rio Grande do Sul, RS, BR.; V Departamento de Ginecologia e Obstetricia, Faculdade de Medicina, Universidade Federal do Ceara, Ceara, CE, BR.

**Keywords:** Maternal Health (MeSH), Metabolomics (MeSH), Translational Medical Research (MeSH), Prediction

## Abstract

The prediction or early diagnosis of maternal complications is challenging mostly because the main conditions, such as preeclampsia, preterm birth, fetal growth restriction, and gestational diabetes mellitus, are complex syndromes with multiple underlying mechanisms related to their occurrence. Limited advances in maternal and perinatal health in recent decades with respect to preventing these disorders have led to new approaches, and “omics” sciences have emerged as a potential field to be explored. Metabolomics is the study of a set of metabolites in a given sample and can represent the metabolic functioning of a cell, tissue or organism. Metabolomics has some advantages over genomics, transcriptomics, and proteomics, as metabolites are the final result of the interactions of genes, RNAs and proteins. Considering the recent “boom” in metabolomic studies and their importance in the research agenda, we here review the topic, explaining the rationale and theory of the metabolomic approach in different areas of maternal and perinatal health research for clinical practitioners. We also demonstrate the main exploratory studies of these maternal complications, commenting on their promising findings. The potential translational application of metabolomic studies, especially for the identification of predictive biomarkers, is supported by the current findings, although they require external validation in larger datasets and with alternative methodologies.

## INTRODUCTION

In addition to the vast knowledge already available in physiology, pathology and therapeutics, there are still some key areas lacking the global and equally spread advantages of health sciences. One of these areas is undoubtedly maternal and perinatal health. Although a significant improvement has been achieved worldwide during the last two decades with the focus provided by the United Nations' Millennium Development Goals (1) and currently by the Sustainable Development Goals (2), the capacity for predicting the most prevalent and hazardous conditions affecting pregnancies, mothers and babies is still very limited. Without prediction, there is no prevention, the pillar for providing good public health. Therefore, generally speaking, there are no available options other than trying to perform an early diagnosis of those potentially harmful conditions, including preterm birth (PTB), preeclampsia (PE), gestational diabetes mellitus (GDM), fetal growth restriction (FGR), maternal and fetal infections and other conditions possibly associated with severe maternal morbidities. However, early diagnosis is almost always more expensive and not truly available for a significant proportion of populations, especially those in low- and middle-income settings.

Considering all these aspects, the purpose of the current review is to summarize the already existing knowledge on technologies that possibly represent new frontiers and future tools for predicting maternal and perinatal conditions responsible for a significant burden of disease in women and children in the world. Metabolomic biomarkers are one of these new promising tools and deserve special attention due to their potentially important role in the management approach for such conditions in the near future.

## METABOLOMICS AS PART OF OMICS TECHNOLOGIES

**Figure 1:**
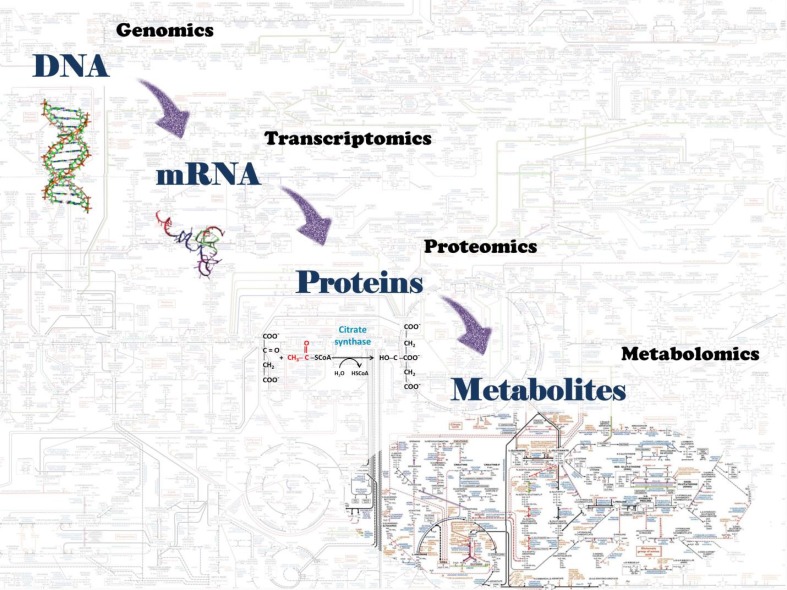
Omics science components of biological systems

The study of the biological systems of an organism leads to the understanding of complex interactions between the genes, their products (RNAs, proteins, and metabolites) and many environmental factors that determine their functioning (3,4). The recent development of what is called systems biology enables the recognition of these integrative pathways. The field of systems biology is related to the capacity to identify thousands of biological molecules and to establish their interactions using advances in bioinformatics, statistics, and high-throughput techniques of sample analysis. The “omics” sciences emerged from this concept of integrative analysis in the field of systems biology and includes genomics, for the study of a set of genes (genome); transcriptomics, for a set of RNAs (transcriptome); proteomics, for a set of proteins (proteome); and metabolomics, for a set of metabolites (metabolome), as shown in Figure 1.

The clinical importance of omics sciences arises from the innovative approach of the dynamic regulations of biological systems, which can be explored by both holistic and reductionist strategies (5). A reductionist strategy is the use of only one part of a complex system or only one hypothesis to build conclusions or to understand the determinants of the whole process. On the other hand, a holistic approach considers multiple interactions and complexities to elucidate the whole system. Prevalent maternal complications, such as PE, PTB, GDM or FGR, are commonly addressed using a reductionist approach. For instance, a woman with a short cervix is considered to have a higher risk for PTB, or a fetus with an estimated weight below the 10^th^ percentile is considered growth restricted. The omics sciences enable discovery-driven studies with a holistic and hypothesis-free approach and may provide the key step forward in addressing complex research problems in maternal and perinatal health, decreasing bias and confounders (3,6–10).

### Genomics

Genomics is the study of genes and their functions and demonstrates the gene codification for proteins that modulate biological systems. Therefore, genomics may be useful to determine the genetic predisposition for a particular disease, identifying the genes involved in its pathophysiology. Genomics studies are more commonly performed using the candidate gene approach, through which preselected genes are sequenced. The complex interactions of genes in the development of a pathologic phenotype and the presence of nongenetic factors limit the results of this approach. Regularly, there is not only one gene involved in the development of a disease but also groups of genes. Moreover, parts of a gene (*locus*) can be related to the development of the disease, while other parts might be related to its prevention, and the polymorphisms and mutations of genes can determine a wide variance in gene expression. The genome-wide sequencing techniques of the exome can demonstrate a more complete view of the genetic predisposition and establish individuals' prognostics and treatment resistance (11,12).

The main potential limitations of genomics are high cost and uncertainty between gene codification and gene expression/phenotype. Only a small portion of the whole genome will be translated, and there are several mechanisms and processes that control the expression of genes (methylation, acetylation, epigenetic mechanisms, imprinting, etc.). Therefore, having the gene codification for a disease does not mean it will develop.

### Transcriptomics

The next step to understand the development of a disease would be the study of gene expression. Transcriptomics is a step-forward technique compared to genomics in terms of downscaling genetic predispositions. Transcriptomics determines mRNA expression using many techniques, such as microarray-based methods, which are used to measure mRNA transcript levels, and sequence-based methods, such as serial analysis of gene expression (SAGE), cap analysis of gene expression (CAGE), massively parallel signature sequencing (MPSS) and RNA-Seq technology (13). RNA-Seq appears to be the most advantageous transcriptomics approach since it requires a small amount of RNA and is a more precise, “clean” (less background signal) and much lower-cost method considering the high-throughput technology (13). The expression of mRNA in a particular tissue can differ according to the timeframe observed. For instance, the expression of proinflammatory proteins during labor is different from that during other pregnancy periods (4,14). Therefore, it is possible to compare the expression of mRNA in a tissue using arrays with tens or hundreds of genes and determine down- or upregulation of the gene expression according to the level of mRNA (4). The overexpression of a gene at a given phase of the biological process related to the development of a particular pathologic condition can uncover key points of the physiopathology of the disease and potential targets for prevention and treatment.

Despite these benefits, transcriptomics faces similar limitations to genomics. There is a recent conceptual discussion against the traditional idea that the final products of our genes are mostly proteins: gene (DNA) → mRNA → protein (15). The main genome-wide sequencing projects have targeted only long protein-coding mRNAs, given this classical interpretation. However, more recent studies have identified many other gene-encoded contributors as long noncoding RNAs that play different roles in biological systems and metabolic pathways but do not encode proteins (16,17). The various mechanisms that take part in the regulation of gene expression, such as chromatin remodeling, adenylation, elongation, splicing, editing, nuclear export, and degradation, may limit the unbiased recognition of all mechanisms between gene expression and the clinical phenotype (15). These mechanisms seem to be extremely complex, requiring more developed research techniques and new approaches for the study of biological systems (5,18,19).

### Proteomics

Proteins are key instruments of biological systems, and their abnormalities can cause or be a consequence of organism dysfunctions. The study of the proteins contained in a sample is called proteomics, which basically includes identification and quantification (3). Proteomics is a promising approach that reflects genetic and environmental effects in the development of pathological conditions. According to an evolutionary hypothesis regarding protein function, there are some proteins involved in the various metabolic pathways of an organism and others related to gene regulation and expression, including intra- and extracellular signaling and the mechanisms of gene expression (20). The abundance of proteins required to perform such roles is different depending on the complexity of their functions. Contrary to what would be obvious, highly specialized biological processes do not require high protein availability. Moreover, protein activity can vary depending on many factors, such as the concentration of substrates and the presence of other coenzymes. Therefore, the abundance of proteins seems not to be a reliable parameter to address their function. In addition to proteomics limitations, isolating proteins from blood remains a difficult task, and there is no single reliable, accurate and reproducible method capable of obtaining all proteomes (20).

### Metabolomics

The metabolic pathway comprises different biochemical reactions occurring in the intracellular or extracellular compartment. Metabolites are the substrates and products of these metabolic reactions, which require enzymes (proteins), minerals, vitamins, and other cofactors. Metabolomics is the study of the set of metabolites of an organism, identifying and quantifying them with higher sensitivity and more reliable reproducibility (21). The set of these small low-weight molecules is called the metabolome, which is a fingerprint of the metabolism at a given time (3,4,7). Therefore, it is possible to understand the metabolism at that moment or, depending on the sample, to understand the metabolism of days to months ago. The hairs, for example, are stable, contain endogenous compounds, and reflect environmental exposure for many months prior (22). Human metabolomic research emerged from previous experience with plants, microbes, and other less complex mammals after years of bioinformatics/statistics advances to address massive output data (3,4,7). Metabolomics in maternal and perinatal health may enable the identification of biomarkers related to maternal and perinatal complications and the understanding of the physiopathology of the most complex and prevalent diseases, such as PE, PTB, GDM, FGR, maternal/fetal infections and other severe maternal morbidities (10,23–25).

**Figure 2:**
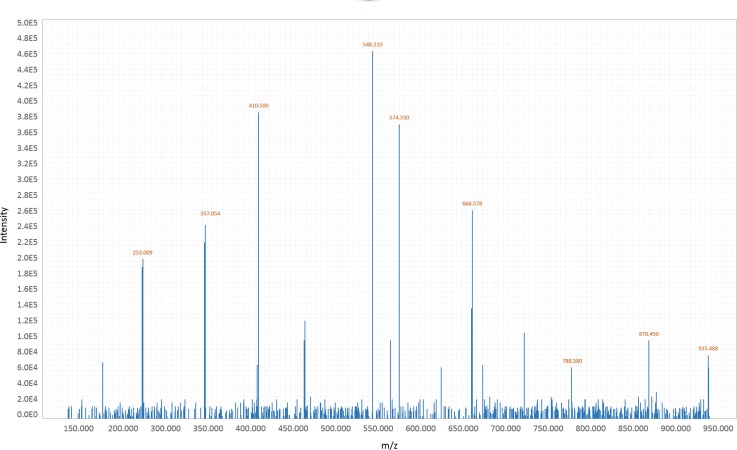
Mass spectrometry spectrum - scheme of metabolite output data.

Advantages of metabolomics include its hypothesis-free and unbiased approach and the downstream result of gene expression, being the closest to the phenotype of the omics sciences. Different technologies have to be employed to identify and quantify fatty acids, bile acids, ketone bodies, amino acids, peptides, carnitines, carbohydrates, vitamins, xenobiotics, steroids, etc. The modalities generally used are gas chromatography-mass spectrometry (GC-MS), liquid chromatography-mass spectrometry (LC-MS) and nuclear magnetic resonance (NMR) spectroscopy. GC-MS and LC-MS can identify and quantify tens of thousands of metabolites at one time, creating substantial output data in mass spectrum format, as shown in Figure 2. Mass spectrometry consists of the following components: 1) Sample inlet, a system that differs between GC-MS and LC-MS; 2) Ion source that ionizes molecules of the sample; 3) Analyzer that separates molecules through a long tube under vacuum according to their mass and charge (using the mass-to-charge ratio – m/z or the time-of-flight method to discriminate different metabolites); 4) Ion detector that detects different metabolites with a sensitivity capable of differing isomeric molecules and measuring the quantity of ions converted into electrical signals. The more molecules that are present, the greater is the electrical signal; and 5) Data analysis system matched to a computer that interprets the signal as a mass spectra data (3,5,9,25). The next step is to identify metabolites using their m/z values and to compare them with their corresponding molecules in a previously known library, namely, the Human Metabolome Database – www.hmdb.ca (26).

There are many different customizations when using metabolomic analytical methods, allowing better identification of a specific range of mass of molecules or metabolites with different solubilities (5,21). The advantages and disadvantages of each method should be considered according to the experimental objectives. NMR methods require minimal sample preparation, preserving the samples in their natural form and can identify metabolites in intact tissues (3-D tissues). However, NMR-based metabolomics has lower sensitivity and requires a greater sample volume. Mass spectrometry-based methods (GC or LC-MS) are usually used as complementary methods, depending on the molecular polarity (polar or nonpolar), sample solubility, and choice for targeted or untargeted metabolomic analyses (3,5,27). The untargeted approach means the study of the whole set of metabolites in a given sample, while the targeted approach refers to the identification of a specific group. The untargeted approach is normally employed to identify new potential predictor biomarkers, and a targeted approach is commonly used for validation analysis to measure previously known metabolites in a given sample (3,9). Especially in untargeted studies, there is a high possibility of returning to the biochemical reactions and to the metabolic pathways with which the identified metabolites are involved. This approach would present an opportunity to address complex diseases that are related to different biological systems (inflammatory, metabolic, energetic, immunologic, etc.).

### Tissues and samples for exploring metabolomics

The use of appropriate biological samples is a key issue in defining the experimental design for metabolomic experiments. Considering pregnancy and childbirth, the available biological tissues can come from the mother (plasma, urine, vaginal fluids, milk, and hair), the fetus/newborn (amniotic fluid, umbilical cord blood, plasma, urine, meconium, saliva and other fluids from the infant), or the placenta. The choice will depend upon each study and the investigating aim defined (3,10).

The most commonly used biological sample is plasma, which is easy to obtain and rich in proteins and metabolites. In biomarker discovery, the final goal is to characterize markers that could enable the early identification of high-risk conditions, and the obvious and most simple screening test would be a blood sample (3,10,23). However, many biomarkers are present in low concentrations in plasma, and its study can be optimized by choosing a biological sample closer to the disease process, especially in the discovery phase, where a great number of identified biomarkers can suggest relevant conditions and pathways involved in the studied diseases (10).

Urine is the second most studied biological sample for metabolomic assays; it is easy to obtain, noninvasive and, like blood, an integrative biofluid. However, the profiling of urine is challenging overall with a wide-ranging variation in metabolite concentrations and fluctuating dilutions due to urine volume alterations. Implementing appropriate statistical analysis methods are pivotal (28).

There is an opportunity for specific metabolomic approaches to study changes in samples closer to diseases or samples clearly influenced by or specific to pregnancy. Vaginal secretions have been studied to understand markers of labor and preterm labor, considering that they can represent changes occurring in the vagina, cervix and adjacent overlying fetal membranes (10,29). There are still challenges in choosing the best way to collect samples and in considering the influences of individual characteristics, protein variations and contamination from previous intercourse or bleeding.

Amniotic fluid (AF) is another potential target for studying metabolomics during pregnancy. Collection of AF depends on an invasive and specialized procedure early during gestation, with potential risks of miscarriage, infection, preterm labor, or bleeding; however, AF is considered to have the best predictive value of metabolic profiling for malformed fetuses (10,30–32). It can also be obtained during labor or prior to delivery, and the discoveries arising could justify and overcome potential risks (33). The use of cord blood has mostly been addressed in neonatal research, with great potential in perinatal asphyxia as a noninvasive method to investigate and understand different conditions in the neonate after delivery (34).

A very important limitation on the use of plasma and most other biofluids is that the metabolites found are greatly dynamic and influenced by diet and immune *status*; furthermore, one isolated sample may not reflect the actual profile. There are standard procedures for sample collection and storage that need to be respected to guarantee accurate results, and immediate processing and freezing are mandatory (3,35,36).

An additional source for metabolomic studies, considering pregnancy and childbirth, is the placenta. There are, however, important considerations regarding how to sample and peculiar difficulties in managing and controlling for confounding factors such as route of delivery, duration of labor, sex of the baby, and the time between delivery and sampling and storage for diverse assays (37). The study of the placenta may play a relevant role in the discovery phase and might help elucidate the involved mechanism and pathophysiology of conditions complicating pregnancy.

The search for biomarkers that can possibly be identified in an easy source of tissue, with no risks during pregnancy, that are noninvasive to obtain and store and, most importantly, that are able to represent long-term metabolite profiles has motivated researchers over the years. Hair metabolomics can provide these advantages and has great potential, with recent interesting results for predicting FGR (22).

### Preterm birth

PTB is defined as childbirth before 37 weeks of gestation, and the real mechanisms of the spontaneous onset of preterm labor/rupture of membranes involved in its occurrence are still unknown. Uterine distension, decidual hemorrhage, stress, autoimmune, infection, inflammation, environmental, behavioral and socioeconomic factors are some of the hypothetical remarkable conditions linked with PTB (38). The identification of risk factors related to PTB has been studied for decades, but the complexity and dynamism of PTB development seem to require a multifactorial approach (4). The attempt to identify biological and sociodemographic markers capable of identifying high-risk pregnant women still has not achieved reasonable results. The only new screening and intervention-based recommendation for PTB prevention discovered in the last two decades is the second-trimester transvaginal measurement of cervical length and vaginal progesterone for women with a short cervix (39,40). Further studies on cervical remodeling and associated biomechanical mechanisms must be performed (41).

Compared to isolated biological markers, the metabolomic approach seems to be superior for demonstrating the organism function (3,4,24,25,38). In 2010, Romero and colleagues published a study evaluating metabolites from processed and stored AF (42). They identified women who had spontaneous preterm labor (PTL) with intact membranes and had been submitted to transabdominal amniocentesis to assess the microbial state of the amniotic cavity and/or fetal lung maturity in three facilities. According to their pregnancy outcomes, women were divided into three groups: 1) with PTL and delivery at term; 2) with PTL, with preterm delivery and without intra-amniotic inflammation/infection (IAI); 3) with PTL, preterm delivery and IAI. Using phase 1 to identify potential metabolites that could differ from subgroups and phase 2 to validate the results of discrimination, the authors demonstrated that the metabolic profile from AF achieved 96.3% and 88.5% accuracy for phase 1 and 2, respectively, to predict the subgroups. Moreover, they also showed differences in the presence of amino acids (AAs) and carbohydrates in the AF samples in the subgroups. The results can contribute to developing AF tests for PTB, to new interventions for high-risk populations or to understanding PTB syndrome.

Menon et al. selected 50 African American women who participated in the Nashville Birth Cohort and collected AF samples during labor: 25 with PTB (before 34 weeks, excluding pPROM) and 25 with full-term births (33). Approximately 350 metabolites were identified in the AF using LC/MS and GC/MS mass analyzers. The metabolites were categorized following their participation in different biochemical pathways, such as histidine, steroids, xanthine, acetaminophen, bile acids, fatty acids, detoxification of xenobiotics and cosmetic-formulation chemical metabolism. The mean predictive accuracy to discriminate PTB from full term was 90%, with 14.2% false negatives. Although the predictive performance of this set of metabolites deserves additional validation, valuable information regarding the possible relationship between maternal liver function and PTB was reported.

In general, the collection of AF is an invasive procedure, and its use for metabolomic analysis is usually reserved when amniocentesis is indicated for another purpose, especially in women with higher risk for spontaneous onset of preterm labor. The reasons must be taken into account to avoid sampling bias, such as risk for fetal malformation or intra-amniotic cavity infection. Graça et al. and Diaz et al. conducted metabolomic studies related to maternal and fetal outcomes, such as GDM, fetal malformations/chromosomal disorders, PTB and pPROM (32,43,45). Urine, blood and AF samples were collected from women at high risk for fetal malformations/chromosomal disorders. Therefore, the different concentrations of allantoin, myo-inositol, alanine, citrate and 2-hydroxyisobutyrate identified in the PTB group might not be generalized.

More recently, Ghartey et al. conducted a nested case-control study inside a prospective cohort to identify cervicovaginal (CV) biomarkers related to spontaneous PTB (29). CV samples were collected at 20 weeks – 23 weeks + 6 days (first visit – V1) and 24 weeks – 27 weeks + 6 days (second visit – V2) gestational age intervals. The CV metabolome was analyzed from 10 women who had spontaneous PTB and 10 women who had term births using ultra-performance liquid chromatography-tandem mass spectrometry (UPLC/MS) and GC/MS. More than 300 metabolites were identified in the CV samples, and women with PTB had a distinct CV metabolome compared with that in samples of women with term birth. Considering V1 samples, more than half of the identified AAs were decreased in PTB samples, and methyl-4-hydroxybenzoate, an antimicrobial agent, was increased in the CV samples of women with term birth. Thus, an increased presence of sialic acid in CV samples of PTB women in V2 samples and downregulation of carbohydrates were identified. Sialic acid plays a role in immune function, specifically on viral entry into the cell. Therefore, there were remarkable differences in CV metabolic markers in women with PTB, enabling the study of some metabolic pathways related to PTB occurrences, such as cervicovaginal protein hydrolysis, inflammation, carbohydrate metabolism and viral/bacterial infection modulation. Metabolomic analyses can be performed using different technical approaches depending on the spectrum of metabolites to be identified. These differences can modify the sensitivity of metabolite identification. Thomas and colleagues conducted a metabolomic analysis to identify discriminatory metabolites in CV fluid in women with PTB and term birth (44). In this study, the identification of AAs, organic acids and fatty acids was prioritized, but the number of metabolites identified was much less than that in Ghartey's study. No significant difference was noticed between the CV metabolites of women with PTB and term birth (44).

Nonetheless, the collection of AF during preterm and term labor brings up a discussion of what benefits each period of sample collection during pregnancy may provide and the possible confounders. There are insufficient data to clarify if different metabolomic profiles during preterm labor are related to the mechanisms of preterm labor determinism or to the proper maternal and/or fetal metabolism that is characteristic of each gestational age period (45,46). A longitudinal targeted metabolomic profile conducted by Lindsay and colleagues showed AAs, nonesterified fatty acids, polar lipids, tricarboxylic acid cycle intermediate metabolic changes in healthy pregnancies (47). HPLC-MS, LC-MS and flow-injection mass spectrometry analyses of plasma samples demonstrated the variance in amino acid concentrations across three set points of pregnancy (at the first, second and third trimester). The sum of the nonbranched chain essential AAs and tricarboxylic acid cycle intermediates increases from the first to the third trimester, whereas free carnitine and acetylcarnitine decrease. The findings seem to be consistent with placental and fetal mediation of AAs during pregnancy, with energetic synthesis and nitrogen cycle changes due to anabolic and catabolic phenomena.

There are complex metabolic cycles mediating fetal development, pregnancy homeostasis, autoimmune regulation, and placental multiple functions. There are many options of samples, a period of collection and metabolomic analyzers to study preterm syndrome, and each one might result in innovative contributions (3,4). The collection of samples during the preclinical phase (asymptomatic women) may provide early identification of higher-risk women as well as lightening trigger mechanisms of preterm labor. Another way to elucidate those triggering mechanisms is collecting samples during labor or from the neonates, demonstrating the resulting PTB metabolic profile (48–50). Multiethnic validation of current findings is required to move forward on preterm prediction and prevention. Therefore, international collaborative studies are essential, shortening the time and financial resources (38).

### Fetal growth restriction

Intrauterine growth restriction is an obstetric disorder characterized by the failure of a fetus to achieve its growth potential (51). It has a multivariate etiology, which includes genetic factors, infections, or uteroplacental insufficiency (52,53). This pathological condition is part of a broad spectrum, composed of “Small for Gestational Age” (SGA) fetuses, which includes all fetuses whose weight is below the 10^th^ percentile for gestational age from the reference ranges applied to the specific population or based on customized charts (51,54). Some of them are physiologically small (constitutional) and therefore are not associated with adverse outcomes (52,53). The incidence of FGR is approximately 4–8% in industrialized countries and 6–30% in low- and middle-income countries (55).

FGR is associated with perinatal complications such as prematurity, fetal death and chronic metabolic disease in adulthood, such as diabetes mellitus type 2, hypertension and metabolic syndrome (56). The prenatal detection of fetuses with FGR is still a challenge in daily obstetric practice, and among low-risk pregnancies, the detection rate is approximately 15% (56). Whereas birth weight is a determinant of mortality and neonatal morbidity, much interest has been raised by research on new and effective means for the early diagnosis and/or prediction of FGR and the improvement of the clinical management (53).

Some single parameters have been tested or, when combined, compose a multifactorial model. The isolated analysis of serum adiponectin in pregnant women between 11 and 13 weeks, performed by Nanda and colleagues in 2011, revealed the inability of that factor to predict FGR because the concentration of adiponectin was the same in both the group with and the group without FGR (57). In an analysis of a multifactorial model, a group in the United Kingdom tested the association of mean maternal blood pressure, nuchal translucency, chorionic gonadotropin, serum pregnancy-associated plasma protein (PAPP-A), pulsatility index of the uterine arteries, placental growth factor (PlGF), placental protein 13, and disintegrin and metalloprotease 12 (ADAM12). This model showed a 73% detection rate for FGR, with a false positive rate of 10% (58). In a second analysis, associating the pulsatility index of the uterine arteries, average maternal blood pressure, PAPP-A, and PlGF, the group achieved a 52.3% detection rate of FGR, with 10% false positives (59). However, the main challenge of this analysis is the great influence imposed by the frequent coexistence between preeclampsia and FGR in their results (60).

More recently, a meta-analysis evaluating the predictive ability of uterine artery Doppler performed in the first trimester achieved a sensitivity of 39.2% for cases of FGR established early (61). In this search for effective screening tools to accurately identify women at the highest risk of FGR, metabolomics has emerged as a new science, seeking biomarkers that can compose a predictive model, which could provide early diagnosis of FGR, with a reduction in morbidity and neonatal mortality. These studies have evaluated the metabolomes of biofluids (urine and blood) or hair for comparison between fetuses with FGR and fetuses with adequate weight for gestational age.

In 2010, Horgan and colleagues studied venous cord blood from women exhibiting the delivery of a healthy singleton fetus and from women with a suspected diagnosis of SGA (birth weight below the 10^th^ percentile for gestational age) (62). Parallel to this analysis, women at 15 weeks of gestation underwent the analysis of plasma samples. Forty women who delivered SGA babies were matched to 40 controls who had uncomplicated pregnancies. The metabolomics of both analyses showed 29 metabolites in very different concentrations between the comparison groups (adequate for gestational age *vs* SGA). Of these, 19 metabolites were identified as predictors of the model and applied to analyses of cord blood and maternal peripheral blood. In the latter case, the predictive model showed an area under the curve (AUC) of 0.9, which represents a robust predictive model of presymptomatic SGA (62).

Dessi and colleagues studied the urine metabolic profiles of neonates with FGR and compared them with controls to define the metabolic patterns associated with this pathological condition. They observed a higher concentration of metabolites such as myo-inositol (also found by Barberini et al. (63), sarcosine, creatine, and creatinine in FGR neonates. An increase in these metabolites in the urine is observed in states of hypercatabolism, as is the case with fasting, and there is a lower level of protein synthesis (64). On the other hand, Maitre and colleagues found decreased levels of tyrosine, acetate, trimethylamine, and formate in maternal urine samples of the late first trimester (65). These molecules can play a role in carbohydrate and fat metabolism (acetate), function as precursors of neurotransmitters (tyrosine), act as mediators of cell death due to enhanced levels of reactive oxygen species (formate), or simply be markers of vegetable intake (trimethylamine). These findings highlight the complexity of FGR pathogenesis and provide some clues that metabolic changes can take place as soon as 11 weeks of gestation. Recently, Sulek and colleagues evaluated the metabolome of hair collected between 26 and 28 weeks from 41 healthy pregnant women whose fetuses developed FGR (birth weight below the 10^th^ percentile for gestational age) and 42 women whose fetuses had adequate birthweight for gestational age. Thirty-two discriminatory metabolites were found, mostly AAs and fatty acids. Five of these metabolites composed a predictive model, which showed an extremely high AUC of 0.998 (22). However, the most severe cases of FGR can be diagnosed as early at 26 weeks of pregnancy, and perhaps 26–28 weeks would not be a suitable interval to screen for FGR in some women.

FGR has a multifactorial etiology, apparently resulting from the interaction between a complex biochemical profile and impaired placental perfusion, which negatively impacts the function of transporting nutrients. The knowledge of metabolism for this condition is still scarce. Therefore, metabolomics could contribute to a better understanding of the pathophysiology of FGR and to a more precise definition of this syndrome. An early detection of fetuses at a higher risk for truly pathological growth restriction or a diagnosis of FGR among pregnant women could improve perinatal outcomes if appropriate interventions can be implemented during prenatal care.

### Pregnancy hypertensive disorders

Preeclampsia (PE) is still a challenging condition in obstetrics practice, involving different clinical manifestations (i.e., early- and late-onset PE) that share the physiopathological aspect of inadequate trophoblast invasion of the maternal vasculature early in pregnancy. There are already known clinical (66) and ultrasonographic risk factors (67): the former can be identified at booking, and the latter can be better screened at the 2^nd^ trimester (uterine artery waveform). Although easily and almost universally accessible, the strongest clinical risk factor history of preeclampsia cannot be applied to nulliparous women, which is a target group for PE (68). In addition, the good performance of uterine artery Doppler velocimetry requires availability of equipment and operator expertise (69). Therefore, great effort has been made to identify biomarkers that could be applied to diagnostic, prediction, or prognostic factors (i.e., soluble endoglin, PIGF and pregnancy-associated plasma protein-A PAPP-A) (70). However, these biomarkers still are not fully and easily available for clinical practice and some of them can only be evaluated at the 2^nd^ or 3^rd^ trimester when there are few therapeutic alternatives for these women.

Considering poor placentation and systemic endothelial activation in PE (71), some authors have been searching for metabolites that could explain the pathogenesis of the disease. Kenny et al. found increased levels of uric acid, which is related to ischemic conditions, and 2-oxoglutarate, or α-ketoglutaric acid, an intermediate of the citric acid cycle (or tricarboxylic acid cycle), which is increased with limited oxidative capacity (72). These metabolites could reinforce the hypothesis of placental hypoxia in preeclampsia, and low levels of taurine could be a marker of defective trophoblast invasion in early-onset PE (71,73).

It is interesting to note that alanine and glutamic acid levels are increased in women already diagnosed with PE (72) and early in pregnancy (74). Both alanine and glutamic acid are nonessential AAs and neurotransmitters. It seems that α-glutamate excitatory effects play an important role in membrane depolarization observed in epileptic seizures (75), which sheds light on the understanding of eclampsia. Alanine represents the main muscle energy source and has an inhibitory action in the brain, as does taurine (75). Renal function may also be affected in PE (76), and augmented levels of creatinine have been observed, either in the 1^st^ (77) or in the 3^rd^ trimester (72).

Omics studies have found many metabolic disturbances in preeclamptic women, such as disorders in carnitine, AAs, carbohydrate or fatty acid pathways, by diverse platforms. However, the available studies have used small sample sizes and do not control for pregnancy complications, such as SGA infants (78), fetal malformations or PTB (31), which can also show metabolic profile alterations. Additionally, it would be enlightening if longitudinal studies could perform data collection at different gestational ages.

On the other hand, it is notable that the AUC, sensitivity, and specificity of predicting early or late PE (71,77) can be improved when regression models mix metabolites with clinical factors, including weight, ethnicity, and mean arterial blood pressure. A first-trimester predictive model that showed promising results, with an AUC of 0.835 in a validation study, enrolled 50 women with early preeclampsia and 108 matched controls (79), which is still a modest number of participants. Therefore, it is fundamental, in the near future, to perform a study involving a heterogeneous group of nulliparous low-risk pregnant women, taking into account that this is the set with the highest risk of preeclampsia. The understanding of the metabolome profile of preeclampsia may contribute not only to prediction but also to improving the knowledge regarding cellular and molecular pathophysiology, providing better management of preeclampsia and, ultimately, better maternal and perinatal outcomes.

### Gestational diabetes mellitus

Gestational diabetes mellitus (GDM) is diabetes that is first diagnosed in the second or third trimester of pregnancy that is not clearly either preexisting type 1 or type 2 diabetes, according to the American Diabetes Association (80). It is related to important maternal, fetal, and neonatal outcomes and is a high-risk factor for diabetes mellitus later in life (80). There are still controversies regarding diagnostic criteria, treatment and monitoring of GDM, but recent advances in omics studies can provide clues about the maternal metabolic profile in normal and diabetic affected pregnancies and may be helpful in understanding and predicting the disease.

Normal pregnancy is characterized by progressive insulin resistance (81) and increased levels of lipoproteins and lipoprotein-cholesterol (82), and both phenomena increase glucose levels. Fat stores can be used as an energy source by the mother, so glucose can reach the fetus, which is the main energy source of the fetal unit and is easily transported through the placenta by facilitated diffusion (81,82). GDM, however, seems to promote a shift from gluconeogenesis to ketone body production, which is probably why fasting acylcarnitine ester levels are lower and 3-hydroxybutyrate are higher in diabetic compared to normal pregnancies (83).

In urine samples of 2^nd^-trimester diabetic pregnancies, Diaz et al. (2011) found increased levels of 2-hydroxyisobutyrate (43). This observation reinforces the association between GDM and type 2 diabetes (81), since this biomarker has also been identified in diabetic patients, reflecting disturbances in free fatty acid metabolism (84). Urinary excretion of 3-hydroxyisovalerate also appears to be increased in GDM (43) and reflects the reduced activity of β-methylcrotonyl-CoA carboxylase, a biotin-dependent enzyme (85). It is important to consider the (1) relationship between previous diabetes and congenital malformation (80) and (2) the possible teratogenic effect of biotin deficiency (85). This finding requires further investigation and may be helpful to improve the diagnostic criteria and follow-up of GDM.

Using proton nuclear magnetic resonance (^1^H-NMR) spectroscopy to study urine samples of 1^st^ and 2^nd^-trimester pregnancies, Sachse et al. (2012) have shown that urine citrate increases with higher degrees of hyperglycemia (86). However, they did not find any difference in the metabolomic profile between normal and affected women, regardless of the diagnostic criteria of GDM, even after normalizing the results to the creatinine level or considering that the urine metabolomic profile is influenced by the immediate lifestyle and diet and less by the genetic background. On the other hand, UPLC-MS identified slightly increased levels of choline in urine in the 2^nd^ trimester of prediagnostic GDM women (31). However, as choline was also altered in fetal malformations and plays a role in fetal brain development, it seems to be nonspecific for GDM (43).

Hair metabolomics was also investigated as a potential marker for GDM. Baker and colleagues analyzed hair metabolites in two different cohorts using samples of women who developed GDM matched by BMI to controls with uncomplicated pregnancies. Analyzing hair samples collected at the time of oral glucose tolerance testing (24–28 weeks of gestation) of 47 Chinese women in each group, the authors found one metabolite (adipic acid) that differs significantly between groups and might be potential discriminatory (87). Using hair samples collected earlier in pregnancy (at 20 weeks) of women who participated in the SCOPE study, two metabolites (itaconic acid and cis-aconitate) had significantly different levels between groups (20 women with GDM and 26 controls) (88). Although the results are from pilot studies, new potential approaches demand investigations in larger datasets.

Studies have used various metabolomic technologies, in either AF, maternal plasma, serum, hair, or urine samples, at various times during pregnancy. Each technique and biological sample has distinct properties of sensitivity, specificity, and power to detect metabolic changes that affect GDM, and findings are still inconclusive (89). A comprehensive overview of this condition could be approached by studies involving several biological fluids in different gestational ages and taking into account dietary intake and treatment options (89). GDM is a worldwide problem, and its assessment during pregnancy can improve gestational and perinatal outcomes. More research is needed to identify metabolites that could be used as biomarkers of disease and to better define this condition.

## Maternal and fetal infections

Metabolomics has also been used for translational research in infectious diseases during pregnancy. The possibility of identifying a pattern of metabolites related to the more severe condition or to the development of fetal/neonatal sequelae, confirming a maternal-fetal transmission and recognizing potential pathological conditions of the fetus is a worthy approach, especially when current diagnostics tests are not sufficiently sensitive and specific. The current spread of Zika virus infection is a very recent example of how challenging the identification of pathophysiological mechanisms and the creation of reliable serological tests in some infectious diseases can be. The laboratory testing for Zika virus infection guidelines of the WHO demonstrates the difficulty in confirming maternal infection for suspected cases with more than one week after onset of symptoms or with a fetal diagnosis of neurological impairment in asymptomatic women (90). The CDC diagnostic testing recommendations highlight the common cross-reaction with other related flaviviruses, such as dengue, chikungunya, and yellow fever viruses (91), calling attention to the importance of more reliable tests.

Recently, Zhou and colleagues demonstrated dynamic changes in the metabolomic profile of mice during *Toxoplasma gondii* infection (92). The authors showed that a set of metabolites could discriminate infected samples from controls, showing an AUC of 0.996. Fanos and colleagues conducted a metabolomic approach using urinary samples of newborns infected by cytomegalovirus and controls (10). The preliminary investigation showed that the abundance of a set of metabolites was significantly different between the groups. Various studies related to nonpregnancy infections, such as *Haemophilus influenzae*, dengue, malaria, tuberculosis, and *Clostridium difficile,* have already demonstrated the potential contribution of metabolomics to the pathogenesis, mechanisms of adaptation, severity, host response and impairment (93–96).

Another potential applicability for metabolomic studies is chorioamnionitis, an infection that can present not only as a subclinical condition with minimum consequences for women or fetuses/newborns but also as a severe infection leading to sepsis and severe maternal and neonatal morbidity and mortality (97–101). Chorioamnionitis is a major cause of mortality in many countries and lacks reliable markers for early diagnosis (97,102,103). For instance, women with PROM, especially if preterm, are at higher risk for infection, and currently available markers collected from blood, vaginal secretions or AF have not proven to be highly accurate in the prediction or early diagnosis of chorioamnionitis (97,102,104–108). In this case, a false positive test for infection can lead to unnecessary interventions, such as prematurity, and a false negative test might determine the development of neonatal sepsis. Therefore, not only a timely identification of infection but a more accurate diagnosis is crucial to prevent severe adverse outcomes.

A few metabolomic studies have been conducted to address the early identification of women with amniotic infection. A metabolomic analysis of AF from 40 women with rupture of membranes or preterm labor was performed, and the results were compared according to the microbiological/histological *status* of infection and neonatal outcomes (infection and perinatal brain injury) (109). Several metabolites of more than 8 metabolic pathways were identified as potential markers of chorioamnionitis. The sphingolipid metabolic pathway was the most important group of metabolites, showing the strongest discriminant power with an AUC of >0.99% and >0.95% to discriminate cases with and without brain injuries in chorioamnionitis cases, respectively. The authors presented a set of metabolites as potential best biomarker candidates, discussing many possible correlations and underlying mechanisms related to chorioamnionitis. However, they also clarified that the external validation of findings is limited due to the use of specific methodology, excluding some phenotypes of women and newborns to create a more homogenous comparison analysis. According to the authors, this homogeneity of outcomes is not what is found “in real life”.

A study from Romero and colleagues also demonstrated that metabolomic profiling is useful for discriminating cases of preterm delivery with and without intra-amniotic infection (42). Another case-control study, this time using lipidomic analysis of the AF, also identified potential markers for amniotic infection (110,111). Both findings still require further validation.

Metabolomic studies assessing potential makers for chorioamnionitis usually use AF samples collected after the rupturing of membranes through amniocentesis in sites where this invasive procedure is generally standard to address amniotic infection. The AF is a key tissue for maternal and perinatal research, considering its interactions with the placenta, mother, and fetal tissues, providing RNA, DNA and metabolites of all three components (24,112). Nevertheless, more studies using blood, urine, hair, or vaginal secretion markers might be important to develop a more reproducible and less invasive method to investigate chorioamnionitis.

### Severe maternal morbidity and other conditions

Maternal complications during pregnancy, childbirth and postpartum periods are part of a continuum or spectrum of morbidity classified from mild to severe. Depending on the severity, the morbidity can be potentially life-threatening and put women at risk for “maternal near miss” or maternal death. Pragmatically, maternal near miss is defined as an experience of near death that can be identified considering aspects of clinical, laboratory and management-based criteria, according to the World Health Organization (113).

Delays in preventing, diagnosing, or treating maternal complications are related to poorer maternal outcomes (114). Therefore, the early identification of severe maternal morbidity might provide a window of opportunity to save mothers from short- and long-term adverse outcomes. The main causes of maternal mortality are related to hemorrhage and hypertensive complications (101), followed by sepsis, abortion, embolism, and other indirect causes. Clinical tools are being studied to identify, measure, and monitor maternal morbidity (113,115,116). Ordinarily, the clinical tools are composed of maternal symptoms and signs and clinical support characteristics. This means that the diagnosis of maternal morbidity occurs in an advanced stage of severity. Despite being a great advance, the use of the definition of potentially life-threatening conditions and maternal near miss would be even more useful to prevent maternal death and short- and long-term adverse consequences, with earlier diagnoses.

The clinical tool called the Sequential Organ Failure Assessment (SOFA) can be used to identify severe maternal morbidity. However, even this well-known and widely used tool demands a certain degree of organ dysfunction as a proxy for prediction. Metabolomic analyses could be an interesting approach to early identify severe maternal morbidity (life-threatening conditions and maternal near miss). The identification of common pathways of preclinical disorders related to maternal morbidity could be useful, widening the window of opportunity to act before severe organ dysfunction. Some studies have already described altered metabolomic profiles related to acute heart failure induced by shock, severe sepsis, and septic and hemorrhagic shock (117–119). The results show that metabolomic profiles can discriminate degrees of severity and may be used for early diagnosis. Therefore, such profiles might be useful to predict organ failure or systemic inflammatory response syndrome in pregnant women as well, independent of the cause of morbidity.

Based on the same idea, the identification of a metabolomic pattern directly related to the main causes of severe maternal morbidity, hypertensive and hemorrhage disorders seems to be tangible. Aberrant placentation, for instance, is related to hypertensive and hemorrhagic complications during pregnancy. The abnormal remodeling of spiral arteries leads to ischemic placental disease mediated by many inflammatory, immunologic, and oxidative stress pathways (120,121). A discovery of metabolomic analyses using samples from women with potentially life-threatening conditions, maternal near miss, and noncomplicated pregnancies might demonstrate whether it is possible to identify common metabolites related to maternal morbidity.

## DISCUSSION

The 21^st^ century has had remarkable innovations in technology, especially in service industries, such as communication and informatics involving smartphones and high-quality wireless networks, changing the way people live and interact. Modification is usually required by the need for new ways to deal with a competitive routine and a large amount of information in different parts of the globe, all of which is part of the new era of globalization phenomena. There is a classic theory about innovations and economic growth based on creative destruction (122). Briefly, one of the bases of this theory is the stationary state, which is the gap period between the development of new research and, consequently, new innovations. This state can lead to innovation mostly depending on the rate of interest and the number and impact of available innovations. Translating this concept to our area of interest, we could say that research on maternal and perinatal health urges innovation.

Maternal and child health are priority topics of the third sustainable development goal (2). Obstetric complications, such as PE, PTB, FGR, and GDM, have a major impact on maternal and perinatal health because they can lead to short- and long-term consequences for women or newborns, from childhood to adulthood. Biological biomarkers seem to be essential for the development of predictive models because they play an important role in the pathophysiology, whether in cause or in consequence mechanisms. The integrated study of the biological interactions in an organism (systems biology) has led to omics-based research, which has been demonstrated to be a promising and useful approach to assess such complex syndromes (3,38). To yield reliable and reproducible information, samples must follow the specific and detailed methodology for processing and storage, and data on these details are key to allow comparisons between studies and advances in metabolomic research in maternal-fetal medicine.

Metabolomics seems to be the most reasonable approach to studying the majority of obstetrical syndromes, considering the costs, confounders, analytical techniques, output data generation and the possibility of addressing final pathways for the underlying mechanisms involved in the occurrence of each complication. Metabolomic studies have generated optimistic results so far, widening the opportunity to explore new methodologies for sample collection, preparation, and analysis. Apart from that, the technique requires validation of the initial findings of pilot studies in larger datasets, using numerous multiethnic population cohorts. Thus, metabolomics might be a sensible way to move forward in the prediction and prevention of maternal complications, achieving a significant impact on people's lives as the 21^st^-century technology revolution may have done.

The current review aimed to present how metabolomics has become a promising approach for maternal and perinatal health research in recent years, showing (especially for a nonexpert audience) the rationale behind this high-technology method in the omics science perspective and its potential translational application. The main maternal complications during pregnancy, such as preeclampsia, PTB, FGR and GDM, have multifactor complex etiologies, and certainly, an expressive number of unknown underlying factors, but the pathways related to the occurrence of these complications are not yet clarified. Such complexity makes their prediction extremely challenging also considering that the pathophysiology might involve genetic and environmental adaptive mechanisms from maternal and fetal components. Metabolomics is the study of the metabolites in a given sample, and it is the closest correspondent to the cell, tissue or organism function compared to other omics sciences, such as genomics, transcriptomics or proteomics. Metabolomic studies of maternal complications during pregnancy can be a valuable method to explore biomarkers in different sources of samples, although advances in reproducible analytical procedures and external validations in larger datasets are still necessary.
